# Hyperexcitability of the local cortical circuit in mouse models of tuberous sclerosis complex

**DOI:** 10.1186/s13041-019-0427-6

**Published:** 2019-01-25

**Authors:** Jian-Ping Zhao, Akira Yoshii

**Affiliations:** 1 Department of Anatomy & Cell Biology, University of Illinois at Chicago, Chicago, IL USA; 2 Department of Brain and Cognitive Science, Massachusetts Institute of Technology, Cambridge, MA USA; 3 McGovern Institute for Brain Research, Massachusetts Institute of Technology, Cambridge, MA USA

**Keywords:** Tuberous sclerosis complex, E/I balance, AMPA receptor, GABA receptor, Autism, Epilepsy, mTOR pathway

## Abstract

Tuberous sclerosis complex (TSC) is a neurogenetic disorder associated with epilepsy, intellectual disabilities, and autistic behaviors. These neurological symptoms result from synaptic dysregulations, which shift a balance between excitation and inhibition. To decipher the synaptic substrate of hyperexcitability, we examined pan-neuronal *Tsc1* knockout mouse and found a reduction in surface expression of a GABA receptor (GABAR) subunit but not AMPA receptor (AMPAR) subunit. Using electrophysiological recordings, we found a significant reduction in the frequency of GABAR-mediated miniature inhibitory postsynaptic currents (GABAR-mIPSCs) but not AMPAR-mediated miniature excitatory postsynaptic currents (AMPAR-mEPSCs) in layer 2/3 pyramidal neurons. To determine a subpopulation of interneurons that are especially vulnerable to the absence of TSC1 function, we also analyzed two strains of conditional knockout mice targeting two of the prominent interneuron subtypes that express parvalbumin (PV) or somatostatin (SST). Unlike pan-neuronal knockout mice, both interneuron-specific Tsc-1 knockout mice did not develop spontaneous seizures and grew into adults. Further, the properties of AMPAR-mEPSCs and GABAR-mIPSCs were normal in both *Pv-Cre* and *Sst-Cre* x *Tsc1*^*fl/fl*^ knockout mice. These results indicate that removal of TSC1 from all neurons in a local cortical circuit results in hyperexcitability while connections between pyramidal neurons and interneurons expressing PV and SST are preserved in the layer 2/3 visual cortex. Our study suggests that another inhibitory cell type or a combination of multiple subtypes may be accountable for hyperexcitability in TSC.

## Introduction

The connectivity between pyramidal cells and interneurons matures during development and is one of the key determinants of critical period plasticity [1–6]. The balance between excitation and inhibition (E/I balance) underlie normal neuronal circuit functions that underlie learning, memory, and sensory processing. Therefore, a deviation of E/I balance toward excitation, or hyperexcitability is implicated in brain disorders including epilepsy and autism spectrum disorders [7–12].

One of the neurogenetic disorders that are associated with seizures and autistic behaviors is tuberous sclerosis complex (TSC) caused by mutations in *TSC1* or *TSC2* [13]. The TSC-1 and -2 proteins form a complex that suppresses the mammalian target of rapamycin (mTOR) pathway, which regulates cell growth, protein synthesis, autophagy and transcription [14]. The mTOR pathway also plays critical roles in synaptic functions [15, 16]. For example, *Tsc2* heterozygous mutant mice have impaired late long-term potentiation (L-LTP) and long-term memory [17]. Hippocampal neurons that are suppressed with *Tsc-1* and *-2* using RNA interference have impaired α-amino-3-hydroxy-5-methyl-4-isoxazolepropionic acid receptor (AMPAR) currents [18]. *Tsc-1*-deleted hippocampal neurons exhibit impaired long-term depression (LTD) mediated by a metabotropic glutamate receptor (mGluR) [19, 20]. Furthermore, an E/I balance is skewed to hyperexcitability in *Tsc-1*-deleted hippocampal neurons [21].

A local cortical circuit consists of pyramidal neurons and inhibitory neurons, which maintain the E/I balance [22–27]. Recent advances in mouse genetics offer an analysis of subpopulations of inhibitory cells based on molecular markers [28, 29]. Among various interneurons, cells expressing parvalbumin (PV) and somatostatin1 (SST) are the most extensively studied. PV(+) cells are either large basket cells that contact with the soma of pyramidal neurons or chandelier cells which project to the axon initiation segment [22, 23, 30–32]. Together, PV (+) cells suppress generation of action potentials in the pyramidal neuron. In the cortex, Martinotti neurons that are positive for SST connect to distal dendrites of a pyramidal neuron [23, 33]. In the rodent visual cortex, PV (+) and SST (+) cells play complementary roles in computing sensory inputs [34–37]. In a mouse model of Rett syndrome, PV (+) and SST (+) interneurons play distinct roles [38–40]. Further, PV (+) interneuron are involved in the pathophysiology of genetic epilepsy syndromes including mutation of the NAV1.1 sodium channel, which is associated with Dravet syndrome [41]. Therefore, it is an intriguing possibility that PV (+) or SST(+) cells play a critical role in hyperexcitability of the local circuit in TSC.

In this study, we examined pan-neuron-specific synapsin1-Cre (*Syn1-Cre*) x *Tsc1*^*fl/fl*^ mice and found that they had reductions in both total and surface expressions of Gamma-aminobutyric acid type A receptor subunit alpha-1 subunit (GABA_A_R-α1). *Syn1-Cre* x *Tsc1*^*fl/fl*^ mice also had a reduction in GABAR-mediated miniature inhibitory postsynaptic currents (GABAR-mIPSCs) in layer 2/3 visual cortical pyramidal neurons, indicating hyperexcitability. To further study the origin of hyperexcitability in TSC, we created PV- and SST- cell-specific *Tsc1* knockout mice. Unlike *Syn1-Cre* x *Tsc1*^*fl/fl*^ mice, both interneuron-specific knockout mice grew into adulthood without spontaneous seizures. In both interneuron-specific mice, electrophysiological recordings of layer 2/3 pyramidal neurons showed normal E/I balance that was comparable to wild-type mice. These results indicate that deleting *Tsc1* in PV (+) or SST (+) interneurons alone does not recapitulate the E/I imbalance that is observed in *Syn1-Cre*) x *Tsc1*^*fl/fl*^ mice. Our study suggests that another or multiple inhibitory cell type(s) may be involved in hyperexcitability that underlies epilepsy and autistic behaviors in TSC.

## Methods

### Animal

This study was carried out in accordance with the principles of the Basel Declaration and recommendations of MIT, UIC and NIH guidelines on the humane care of animals. The protocol was approved by the MIT- and UIC-IACUC. All animal manipulations were approved by the MIT- and UIC-IACUC and were performed in accord with its guidelines. Animals were kept under 12 h light/dark cycle. Neuronal subtype-specific deletion of *Tsc1* was achieved by crossing cell type-specific Cre-driver lines (*PV-Cre,* https://www.jax.org/strain/017320; *Sst-IRES-Cre*, https://www.jax.org/strain/013044; and *Syn1-Cre* https://www.jax.org/strain/003966) with *Tsc1*^*fl / fl*^ mice (https://www.jax.org/strain/005680) [42]. All strains were obtained from Jackson Laboratory. Floxed-*Tsc1* mice served as controls. All mice belonged to the C57BL6/J strain. In all experiments, both male and female animals were used.

### Gel electrophoresis, quantitative immunoblotting, and statistical analysis

**Fig. 1:**
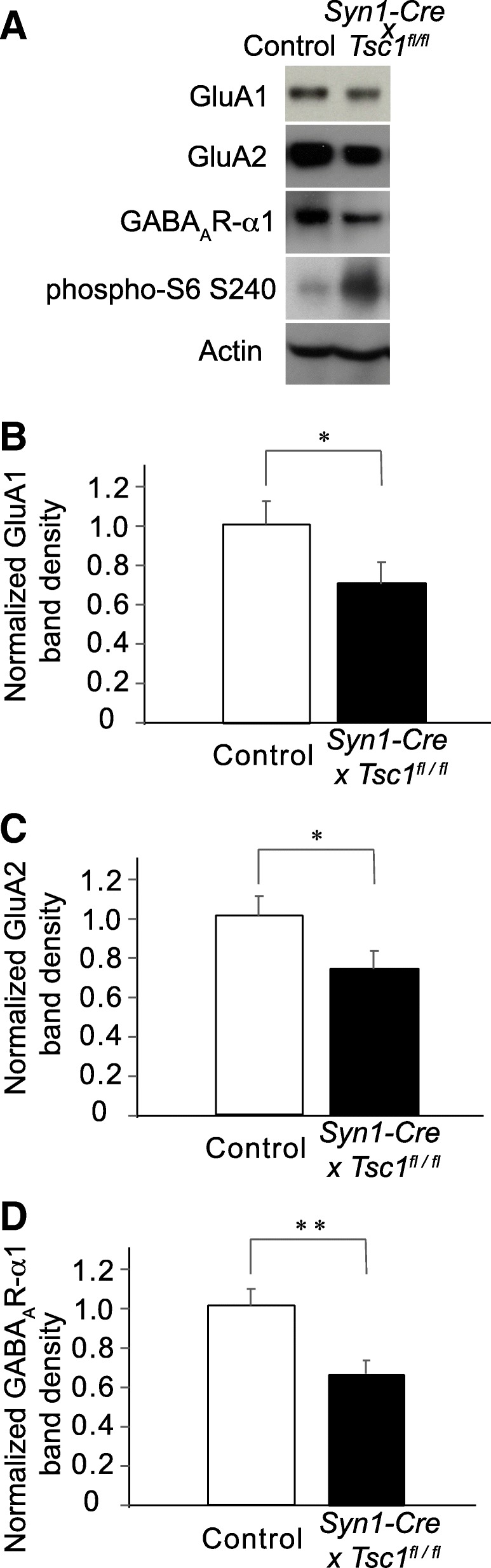
Immunoblots of whole lysates from *Syn1-Cre* x *Tsc1*^*fl/fl*^ mouse visual cortex. **a**. Representative immunoblots of GluA1, GluA2, GABA_A_R-α, phosphor-S6 and transferrin receptor are shown. Whole lysates were prepared from the visual cortex at P28. An increase in phosphor-S6 level indicates the overactivation of the mTOR pathway in a *Syn1-Cre* x *Tsc1*^*fl/fl*^ mouse brain. **b**, **c**, **d** Band intensities of GluA1, GluA2 and GABA_A_R-α are normalized to actin. The mutant brains showed significant reductions in GluA1, GluA2 and GABA_A_R-α band intensities. * *P* < 0.05 in (**b**) and (**c**). ** *P* < 0.01 in (**d**). Error bars represent s.e.m. in (**b**), (**c**) and (**d**)

**Fig. 2:**
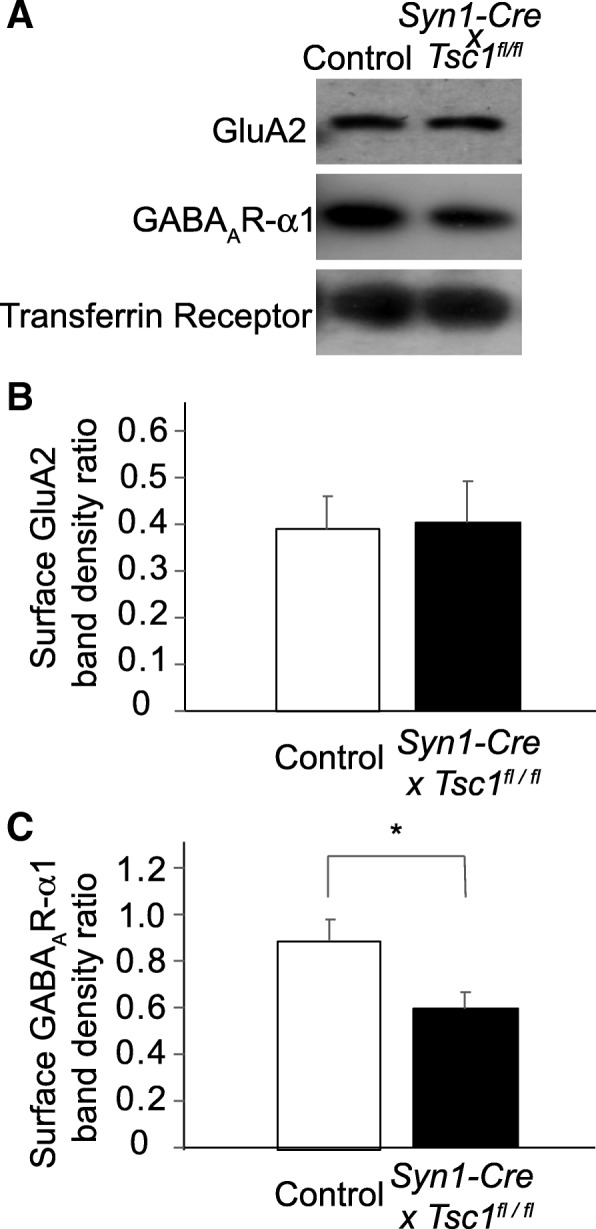
Surface biotinylation assay of dissociated *Syn1-Cre* x *Tsc1*^*fl/fl*^ mouse cortical neurons. **a** Surface receptors were biotinylated in cultured *Syn1-Cre* x *Tsc1*^*fl/fl*^ mouse cortical neurons and pulled down using neutravidin-agarose slurry. Representative immunoblots of GluA2, GABA_A_R-α and transferrin receptor are shown. **b**, **c** Band intensities of GluA2 and GABA_A_R-α are normalized to the transferrin receptor. While the mutant neurons showed GluA2 band intensity ratio comparable to control, they had a significant reduction in GABA_A_R-α band intensity ratio. * *P* < 0.05 in (**c**). Error bars represent s.e.m. in (**b**) and (**c**)

Concentrations of protein sample were measured using Pierce BCA Protein Assay Kit (Thermo Scientific). Equal amounts of proteins (5 or 10 μg per lane) were run on 6 or 8% SDS-PAGE, then transferred to polyvinylidene difluoride (PVDF) membrane by electroblotting (Idea Scientific, Minneapolis, MN). Blots were blocked with blocking buffer (Sigma) diluted with Tween/0.1 M PBS (TPBS) for 30 min, then incubated in primary antibody in TPBS for 1 h at room temperature. The following primary antibodies were used; GluA1 (Millipore Cat# 05–855, RRID:AB_10015249), GluA2 (Millipore, Cat#MAB397, RRID:AB_2113875), GABA_A_R-α1 (NeuroMab, clone N95/35, RRID:AB_10697873), actin, phosphorylated S6 (Cell Signaling Technology, #4858, RRID:AB_916156), transferrin receptor (Santa Cruz, #sc-9099, RRID:AB_2201346) and β-actin-HRP (Thermo Fisher Scientific Cat# MA5–15739-HRP, RRID:AB_2537667). After three 5 min rinses in TPBS, blots were incubated in secondary antibody (horseradish-peroxidase-conjugated goat anti-rabbit or goat anti-mouse (Jackson ImmunoResearch) in TPBS at 1:5000 or 1:10,000), washed several times for 5 min in TPBS, reacted with chemiluminescent substrate (Pierce, Rockford, IL), and exposed to film (Kodak, Rochester, NY). Films were scanned, and band density was measured by Image J. Measurements were confirmed to be within the linear range of density analyses of dilution series of the samples. Band intensities of GluA2 and GABA_A_R-α1 subunit were normalized to actin in Fig. 1 and to transferrin receptor in Fig. 2. Results are reported as averages + the standard errors of the mean (s.e.m.). All immunoblot analyses used three sets of protein samples from three different litters. Two gels were run for each sample and blotted (*N* = 6).

### Primary neuron culture and biotinylation assay

Occipital cortices of mouse brains were dissected at embryonic day 15.5 (E15.5), digested with a solution containing papain and DNase for 25 min. The rest of fetal brain tissues were used for DNA extraction followed by genotyping. Cells were dissociated using fire polished glass pipets and plated to a coverslip coated with laminin and poly-D-lysine. On day in vitro (DIV) 18, a cell surface biotinylation assay was performed. Neurons were preincubated with tetrodotoxin (2 μM) with leupeptin (200 μg/ml) for 30 min at 37 °C to avoid lysosomal degradation of internalized receptors. Cultures were serially cooled to 10 °C and 4 °C to stop membrane trafficking in cold DPBS plus 1 mM MgCl_2_ and 2.5 mM CaCl_2_, then treated with cleavable (disulfide-linked) biotin containing solutions (1.5 mg/ml sulfo-NHS-biotin in DPBS) for 30 min to label surface receptors. Unbound biotin was quenched with cold DPBS plus 1 mM MgCl_2,_ 2.5 mM CaCl_2_ and 50 mM glycine (rinse, 2 × 5 min). Cells were lysed for 3 min in 0.5% SDS-RIPA buffer [1% Triton-X-100, 0.1% SDS, 0.5% deoxycholic acid with (in mM) 50 Tris pH 7.4, 150 NaCl, 2 EDTA, 50 NaF, 1 sodium orthovanadate, and protease inhibitor cocktail] and scraped. The lysed solution was centrifuged at 16,000x g for 15 min at 4 °C. Protein concentration of the supernatant was determined and 300 μg of protein was adjusted to a volume of 1 mL with 0.1% SDS-RIPA buffer. Biotinylated receptor subunits were precipitated with 70 μl neutravidin-agarose (ThermoFisher, IL) slurry overnight at 4 °C, washed with the SDS-RIPA buffer and spun down three times. Pulled-down subunits were eluted into Laemmli sample buffer by boiling for 6 min. Biotinylated receptor subunits were detected by immunoblot and were analyzed by normalizing the band intensity to that of transferrin receptor.

### Electrophysiology

Young (P28–P30) male floxed and conditional *Tsc1* knockout mice were anesthetized with isoflurane and decapitated. The brain was quickly removed and placed in ice-cold artificial cerebrospinal fluid (ACSF) containing the following (in mM): 124 NaCl, 2 MgCl2, 3 KCl, 3 CaCl2, 1.25 NaHPO4, 26 NaHCO3, and 25 D-glucose saturated with 95%O_2_/5% CO_2_ to a final pH of 7.35. Coronal VC slices (280 ~ 300 μm) were cut in ice-cold ACSF with a vibratome (Leica 1000-plus sectioning system) and incubated in at 30–32 °C ACSF for at least 30 min then at room temperature (23 ~ 25 °C) for one hour for recovery.

Individual slices were transferred to a submersion recording chamber and continuously perfused with carbogenated ACSF at 3–4 ml/min at room temperature, voltage-clamp whole-cell patch recordings were performed from pyramidal neurons at layer 2/3 of the VC, which were visualized by a Nikon (Tokyo, Japan; E600FN) infrared differential interference contrast (DIC) microscope with a 40x water-immersion objective and CoolSNAP EZ CCD camera (Photometrics, Tucson, AZ). Patch pipettes (3–5 MΩ resistance) were pulled from borosilicate glass (outer diameter, 1.2 mm; inner diameter, 0.69 mm) and filled with internal solution containing (in mM): 122.5 Cs-gluconate, 17.5 CsCl, 10 HEPES, 0.2 Na-EGTA, 4 ATP-Mg, 0.4 GTP-Na, and 8 NaCl, pH 7.3 and osmolarity ~ 290 mmol/kg. pClamp 10.2 (Molecular Devices) was used for data acquisition. Electrical signals were amplified with an Axopatch 200B amplifier, digitized with a Digidata 1322A interface (Molecular Devices), filtered at 2 kHz and sampled at 10 kHz. Chemicals were applied by addition to the ACSF perfusing the slices.

AMPAR-mEPSCs and GABAR-mIPSCs were separately recorded from the same cells in the presence of 1 μM tetrodotoxin and 50 μM D-2-Amino-5-phosphonopentanoic acid (AP5, NMDAR antagonist) when the recorded cell membrane voltage was clamped at − 40 mV and 0 mV. To verify the miniature currents obtained at − 40 mV and 0 mV are AMPAR-mEPSCs and GABAR-mIPSCs only, 6 μM 2,3-dihydroxy-6-nitro-7-sulfamoyl-benzo [f]quinoxaline-2,3-dione (NBQX, AMPAR antagonist) was present in ACSF at − 40 mV clamped voltage and 6 μM Bicuculline methochloride (BMC, GABA_A_ receptor antagonist) at 0 mV clamped voltage in control experiments, they blocked all miniature currents at − 40 mV and 0 mV clamped voltage respectively.

Mini version 6.0 (Synaptosoft, Decatur, GA) was used for data analysis, all miniature currents with amplitudes two times greater than baseline noise were collected over a 3 min period and averaged, averaged miniature currents were measured for amplitudes from peak to noise midline.

### Statistical analysis

Data were expressed as means ± SEMs, n was given as the number of cells / the number of animals. Statistical significance was evaluated by unpaired, two-tailed *t-*test. *p < 0.05, ** *p* < 0.01.

## Results

### E/I balance is skewed toward hyperexcitability in *Syn1-Cre* x *Tsc1*^*fl/fl*^ mouse

To study a synaptic dysregulation, we chose to study neuron-specific *TSC1* knockout mice by crossing *Tsc1*^*fl/fl*^ and *Synapsin1-Cre* (*Syn1-Cre*) strains [42]. We collected whole lysates from the visual cortices of *Syn1-Cre* x *Tsc1*^*fl/fl*^ mice at postnatal day (P) 28 and performed immunoblots. As an indicator of successful suppression of TSC1 in the mutant brain, we confirmed an increase in phosphorylation of a ribosomal protein S6 (Fig. 1a) indicating hyperactivation of mTOR. We found reductions in the total amounts of the GluA1 (Fig. 1a and b: *p* = 0.011) and GluA2 subunits (Fig. 1a and b: *p* = 0.014) of AMPARs and GABA_A_R-α1 subunit (Fig. 1a and c: *p* = 0.0028) in *Syn1-Cre* x *Tsc1*^*fl/fl*^ mice as compared to *Tsc1*^*fl/fl*^ without the Cre recombinase.

While we anticipated biochemical evidence for hyperexcitability, the examination of whole lysates indicates reductions in both the excitatory and inhibitory receptors. Thus, we compared surface expressions of the receptor subunits using a biotinylation assay. We dissected occipital cortical tissues from each fetus at E15.5 and genotyped them. At DIV 18, dissociated neuronal cultures were incubated with biotin, and coated receptors on the cell surface were pulled down with streptavidin-coated beads. We found comparable levels of GluA2 subunits in both control and *Syn1-Cre* x *Tsc1*^*fl/fl*^ neurons (Fig. 2a and b: *p* = 0.87) and a significant reduction of GABA_A_R-α1subunit in *Syn1-Cre* x *Tsc1*^*fl/fl*^ neurons as compared to control (Fig. 2a and c: *p* = 0.011).

**Fig. 3:**
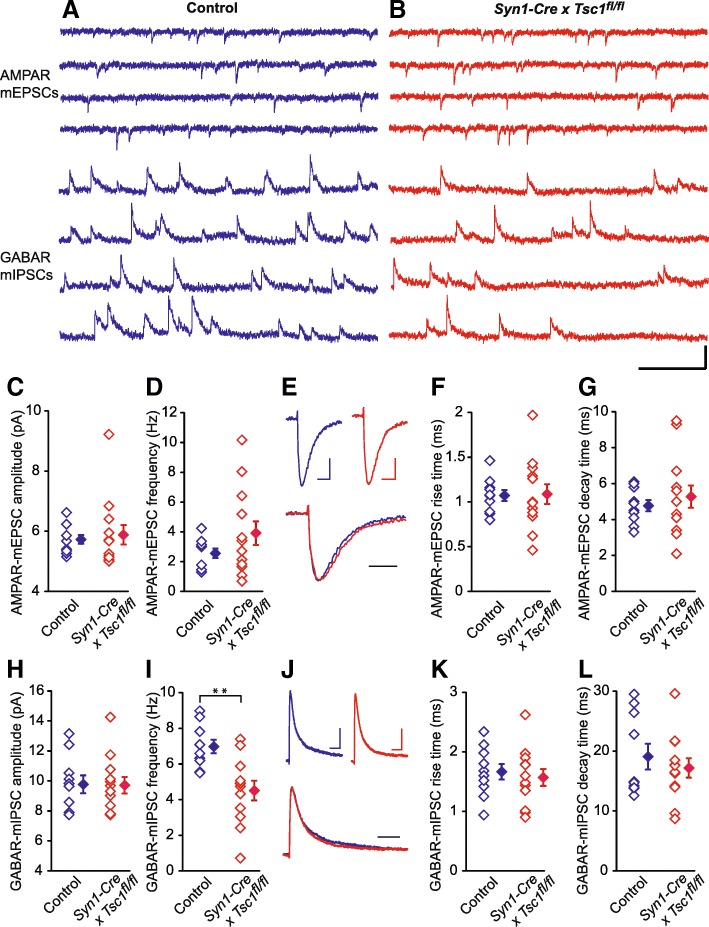
Electrophysiological recordings from layer 2/3 pyramidal neurons in P28–30 control and *Syn1-Cre x Tsc1*^*fl/fl*^ mouse visual cortex. **a**-**b** Sample traces of AMPAR-mEPSC recorded at − 40 mV and of GABAR-mIPSC recorded at 0 mV from same pyramidal neurons in control (blue traces, *Tsc1*^*fl/fl*^ mouse without Cre) and *Syn1-Cre x Tsc1*^*fl/fl*^ (red traces) mice. Scale bar: 20 pA, 200 ms. **c**-**d** Pooled data showing no significant differences in averaged AMPAR-mEPSC amplitude (**c**, Control, 5.73 ± 0.15 pA vs. *Syn1-Cre x Tsc1*^*fl/fl*^, 5.88 ± 0.32 pA, *P* = 0.79) and frequency (**d**, Control, 2.55 ± 0.32 Hz vs. *Syn1-Cre x Tsc1*^*fl/fl*^, 3.91 ± 0.79 Hz, *P* = 0.13) between the mutant and control animals. **e** Top, averaged AMPAR-mEPSCs obtained from control (blue, shown in **a**) and *Syn1-Cre x Tsc1*^*fl/fl*^ (red, shown in **b**) mice, bottom, normalization and superposition of the above averaged AMPAR-mEPSCs. Scale bars: 2 pA, 4 ms. **f**-**g** Pooled data showing no significant differences in averaged AMPAR-mEPSC rise time (**f**, Control, 1.07 ± 0.06 ms vs. *Syn1-Cre x Tsc1*^*fl/fl*^, 1.09 ± 0.11 ms, *P* = 0.95) and decay time (**g**, Control, 4.77 ± 0.31 ms vs. *Syn1-Cre x Tsc1*^*fl/fl*^, 5.28 ± 0.62 ms, *P* = 0.56) between the mutant and control animals. **h**-**i** Pooled data showing no significant differences in averaged GABAR-mIPSC amplitude (H, Control, 9.77 ± 0.6 pA vs. *Syn1-Cre x Tsc1*^*fl/fl*^, 9.71 ± 0.55 pA, *P* = 0.95) and significant difference in averaged GABAR-mIPSC frequency (I, Control, 7.0 ± 0.38 Hz vs. *Syn1-Cre x Tsc1*^*fl/fl*^, 4.5 ± 0.55 Hz, *P* = 0.002) between the mutant and control animals. ** *P* < 0.01. **j** Top, averaged GABAR-mIPSCs obtained from control (blue, shown in A) and *Syn1-Cre x Tsc1*^*fl/fl*^ (red, shown in B) mice, bottom, normalization and superposition of the above averaged GABAR-mIPSCs. Scale bars: 4 pA, 20 ms. **k**-**l** Pooled data showing no significant differences in averaged GABAR-mIPSC rise time (K, Control, 1.67 ± 0.13 ms vs. *Syn1-Cre* x *Tsc1*^*fl/fl*^, 1.57 ± 0.14 ms, *P* = 0.63) and decay time (L, Control, 19.8 ± 2.14 ms vs. *Syn1-Cre x Tsc1*^*fl/fl*^, 17.17 ± 1.62 ms, *P* = 0.48) between the mutant and control animals. For pooled data, unpaired two-tailed *t-*test was used, the open diamonds represent individual experiments and the filled diamonds are means of the experiments in groups, *n* = 10/3 for control and *n* = 13/3 for *Syn1-Cre* x *Tsc1*^*fl/fl*^ groups

To further study the E/I imbalance in the *Syn1-Cre* x *Tsc1*^*fl/fl*^ mouse brain, we used acute visual cortical slices at P28–30 and recorded AMPAR-mEPSCs and GABAR-mIPSCs in layer 2/3 pyramidal neurons (Fig. 3a and b; 10 *Tsc1*^*fl/fl*^ without Cre and 13 *Syn-Cre* x *Tsc1*^*fl/fl*^ cells from three mice). There was no significant difference in AMPAR-mEPSCs amplitude (Fig. 3c: *p* = 0.79) and frequency (Fig. 3d: *p* = 0.13) between control and *Syn-Cre* x *Tsc1*^*fl/fl*^ neurons. We also analyzed kinetics of AMPAR-mEPSCs (Fig. 3e), and both rise time (Fig. 3f: *p* = 0.95) and decay time (Fig. 3g: *p* = 0.56) were comparable between the two strains. While the mIPSC amplitude was not different (Fig. 3h: *p* = 0.95), the frequency was significantly reduced in *Syn-Cre* x *Tsc1*^*fl/fl*^ as compared to control (Fig. 3i: *p* = 0.002). Analyses of GABAR-mIPSC kinetics (Fig. 3j) showed no significant differences in rise time (Fig. 3k: *p* = 0.63) and decay time (Fig. 3l: *p* = 0.48).

A decrease in GABAR-mIPSCs frequency indicates that layer 2/3 pyramidal neurons have fewer GABAergic synapses in the *Syn1-Cre* x *Tsc1*^*fl/fl*^ mouse visual cortex than *Tsc1*^*fl/fl*^ without Cre. No significant alteration in the GABAR-mIPSC amplitude suggests that the remaining inhibitory synapses are likely to be intact. Collectively, both biochemical and electrophysiological analyses indicate hyperexcitability of *Syn-Cre* x *Tsc1*^*fl/fl*^ neurons.

### Analysis of *Pv-Cre* x *Tsc1*^*fl/fl*^ mice

There are various types of interneurons that are classified based on morphologies, molecular markers or electrophysiological properties, and each subclass play a different in cortical computation that is complementary to each other [22–27]. Therefore, each interneuron subtype is likely to contribute to a distinct aspect of pathophysiology that is associated with hyperexcitability [43, 44]. The above results motivated us to ask what interneuron subtype is especially susceptible to the lack of TSC1 function and is accountable for hyperexcitability in layer 2/3 pyramidal neurons of the *Syn1-Cre* x *Tsc1*^*fl/fl*^ mouse visual cortex.

**Fig. 4:**
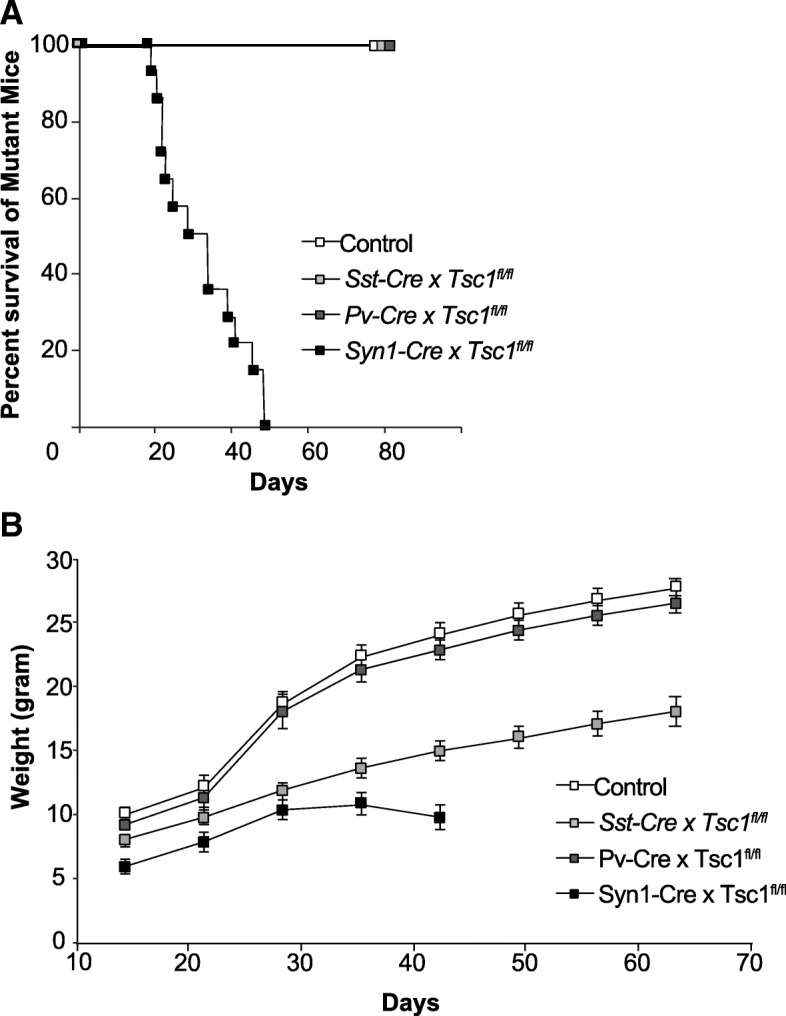
*Syn1-Cre* x *Tsc1*^*fl/fl*^ mice die prematurely but *Pv-Cre* x *Tsc1*^*fl/fl*^ and *Sst-Cre* x *Tsc1*^*fl/fl*^ mice grow into the adult. **a**. Survival curves of the three different conditional knockout mice and *Tsc1*^*fl/fl*^ mouse without Cre (*N* = 15 for each strain). **b**. Weight growth curve. Animals were weighed weekly since P14

**Fig. 5:**
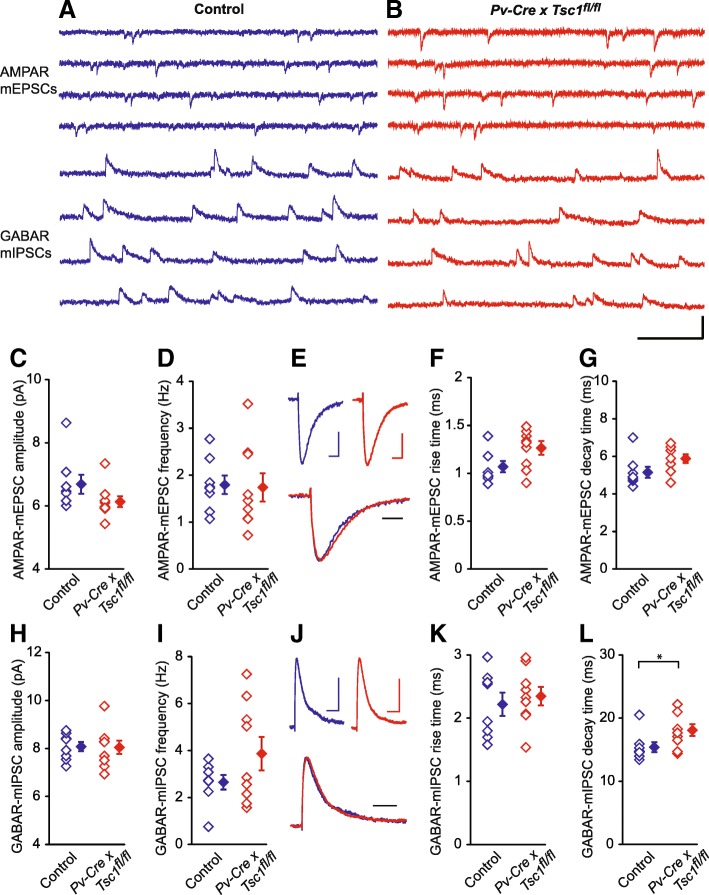
Electrophysiological recordings from layer 2/3 pyramidal neurons in P29 control and *Pv-Cre x Tsc1*^*fl/fl*^ mouse visual cortex. **a**-**b** Sample traces of AMPAR-mEPSC recorded at − 40 mV and of GABAR-mIPSC recorded at 0 mV from same pyramidal neurons in control (blue traces, *Tsc1*^*fl/fl*^ mouse without Cre) and *Pv-Cre x Tsc1*^*fl/fl*^ (red traces) mice. Scale bar: 20 pA, 200 ms. (C-D) Pooled data showing no significant differences in averaged AMPAR-mEPSC amplitude (C, Control, 6.69 ± 0.3 pA vs. *Pv-Cre x Tsc1*^*fl/fl*^, 6.13 ± 0.17 pA, *P* = 0.12) and frequency (D, Control, 1.79 ± 0.2 Hz vs. *Pv-Cre x Tsc1*^*fl/fl*^, 1.74 ± 0.3 Hz, *P* = 0.88) between the mutant and control animals. **e** Top, averaged AMPAR-mEPSCs obtained from control (blue, shown in **a**) and *Pv-Cre x Tsc1*^*fl/fl*^ (red, shown in **b**) mice, bottom, normalization and superposition of the above averaged AMPAR-mEPSCs. Scale bars: 2 pA, 4 ms. **f**-**g** Pooled data showing no significant differences in averaged AMPAR-mEPSC rise time (F, Control, 1.07 ± 0.06 ms vs. *Pv-Cre x Tsc1*^*fl/fl*^, 1.27 ± 0.07 ms, *P* = 0.06) and decay time (G, Control, 5.15 ± 0.29 ms vs. *Pv-Cre x Tsc1*^*fl/fl*^, 5.88 ± 0.23 ms, *P* = 0.07) between the mutant and control animals. **h**-**i** Pooled data showing no significant differences in averaged GABAR-mIPSC amplitude (H, Control, 8.08 ± 0.19 pA vs. *Pv-Cre x Tsc1*^*fl/fl*^, 8.05 ± 0.28 pA, *P* = 0.93) and frequency (I, Control, 2.65 ± 0.31 Hz vs. *Pv-Cre x Tsc1*^*fl/fl*^, 3.86 ± 0.71 Hz, *P* = 0.14) between the mutant and control animals. **j** Top, averaged GABAR-mIPSCs obtained from control (blue, shown in A) and *Pv-Cre x Tsc1*^*fl/fl*^ (red, shown in B) mice, bottom, normalization and superposition of the above averaged GABAR-mIPSCs. Scale bars: 4 pA, 20 ms. **k**-**l**) Pooled data showing no significant difference in averaged GABAR-mIPSC rise time (K, Control, 2.22 ± 0.18 ms vs. *Pv-Cre x Tsc1*^*fl/fl*^, 2.35 ± 0.15 ms, *P* = 0.59) and significant difference in averaged GABAR-mIPSC decay time (L, Control, 15.4 ± 0.78 ms vs. *Pv-Cre* x *Tsc1*^*fl/fl*^, 18.1 ± 0.93 ms, *P* = 0.04) between the mutant and control animals. For pooled data, unpaired two-tailed *t-*test was used, the open diamonds represent individual cells and the filled diamonds are means of the experiments in groups, *n* = 8/2 for control and *n* = 9/2 for *Pv-Cre x Tsc1*^*fl/fl*^ groups

The PV (+) neuron is an attractive candidate since it is implicated in some neurogenetic disorders associated with epilepsy and autism [38–41, 45]. Therefore, we generated *PV-Cre* x *Tsc1*^*fl/fl*^ mouse. Unlike *Syn-Cre* x *Tsc1*^*fl/fl*^ mice, the mutant animals gained weight and lived to become adults (Fig. 4a and b). Further, *PV-Cre* x *Tsc1*^*fl/fl*^ mice did not exhibit dorsiflexion of the tail (Straub tail), humped posture, and hindlimb clasping that were observed in *Syn-Cre* x *Tsc1*^*fl/fl*^ mice [42]. The mutant animals did not exhibit seizures either spontaneously nor with handling. The inducible seizures are characteristic of *Syn-Cre* x *Tsc1*^*fl/fl*^ mice and can be triggered by the gentle spinning of their tails [42]. These seizures consist of a brief behavioral arrest followed by several seconds of clonic activity and tonic extensor posturing of trunk and limbs leading to death. In comparison to *Syn1-Cre* x *Tsc1*^*fl/fl*^ mice, this type of seizure was never observed in *Pv-Cre* x *Tsc1*^*fl/fl*^ mice (*N* = 5). Next, we recorded AMPAR-mEPSCs and GABAR-mIPSCs from nine neurons in two *Pv-Cre* x *Tsc1*^*fl/fl*^ mice and eight neurons in two *Tsc1*-floxed without *Cre* (Fig. 5 a and b), and found no significant differences in amplitude and frequency of mEPSCs (Fig. 5 c and d; *p* = 0.12 and 0.88, respectively) between *Pv-Cre* x *Tsc1*^*fl/fl*^ and control mice. We also analyzed kinetics of AMPAR-mEPSCs (Fig. 5e-g), and both rise time (Fig. 5f: *p* = 0.06) and decay time (Fig. 5g: *p* = 0.07) were comparable between the two strains.

As for mIPSCs, neither amplitude nor frequency were significantly different between *Pv-Cre* x *Tsc1*^*fl/fl*^ and control mice (Fig. 5 h and i; *p* = 0.93 and 0.14, respectively). We also analyzed GABAR-mIPSC kinetics (Fig. 5j-l). The rise time was similar betweebn the two strain (Fig. 5k: *p* = 0.59). However, the averaged decay time was slightly but significantly longer in *Pv-Cre* x *Tsc1*^*fl/fl*^ mouse neurons (Fig. 5l: *p* = 0.04). This finding may result from an altered subunit composition and may suggest a delayed developmental subunit switch or pathological receptor regulation.

These results suggest that the connection between PV (+) interneuron and layer II/III pyramidal neurons does not account for the E/I imbalance that was observed in a cortical circuit of *Syn-Cre* x *Tsc1*^*fl/fl*^ mouse.

### Analysis of *Sst-Cre* x *Tsc1*^*fl/fl*^ mice

**Fig. 6:**
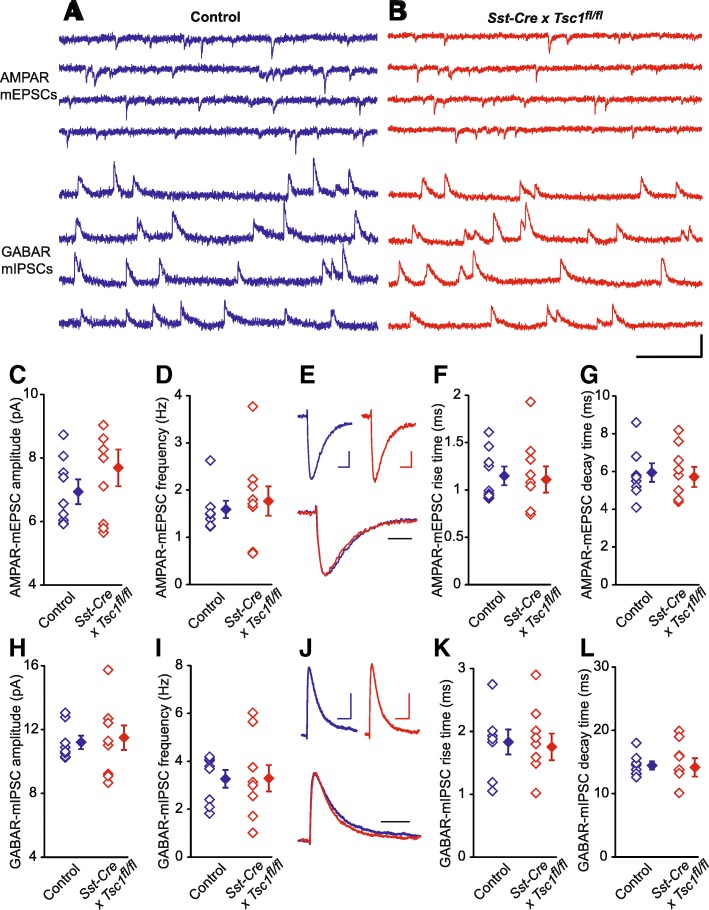
Electrophysiological recordings from layer 2/3 pyramidal neurons in P29 control and *Sst-Cre x Tsc1*^*fl/fl*^ mouse visual cortex. **a**-**b** Sample traces of AMPAR-mEPSC recorded at − 40 mV and of GABAR-mIPSC recorded at 0 mV from same pyramidal neurons in control (blue traces, *Tsc1*^*fl/fl*^ mouse without Cre) and *Sst-Cre x Tsc1*^*fl/fl*^ (red traces) mice. Scale bar: 20 pA, 200 ms. **c**-**d** Pooled data showing no significant differences in averaged AMPAR-mEPSC amplitude (**c**, Control, 6.93 ± 0.39 pA vs. *Sst-Cre* x *Tsc1*^*fl/fl*^, 7.69 ± 0.58 pA, *P* = 0.28) and frequency (**d**, Control, 1.59 ± 0.18 Hz vs. *Sst-Cre* x *Tsc1*^*fl/fl*^, 1.77 ± 0.31 Hz, *P* = 0.66) between the mutant and control animals. **e** Top, averaged AMPAR-mEPSCs obtained from control (blue, shown in **a**) and *Sst-Cre x Tsc1*^*fl/fl*^ (red, shown in **b**) mice, bottom, normalization and superposition of the above averaged AMPAR-mEPSCs. Scale bars: 2 pA, 4 ms. **f**-**g** Pooled data showing no significant differences in averaged AMPAR-mEPSC rise time (F, Control, 1.15 ± 0.1 ms vs. *Sst-Cre x Tsc1*^*fl/fl*^, 1.11 ± 0.14 ms, *P* = 0.82) and decay time (G, Control, 5.94 ± 0.49 ms vs. *Sst-Cre x Tsc1*^*fl/fl*^, 5.71 ± 0.54 ms, *P* = 0.74) between the mutant and control animals. **h**-**i** Pooled data showing no significant differences in averaged GABAR-mIPSC amplitude (H, Control, 11.2 ± 0.42 pA vs. *Sst-Cre x Tsc1*^*fl/fl*^, 11.5 ± 0.77 pA, *P* = 0.74) and frequency (I, Control, 3.26 ± 0.37 Hz vs. *Sst-Cre x Tsc1*^*fl/fl*^, 3.29 ± 0.55 Hz, *P* = 0.97) between the mutant and control animals. **j** Top, averaged GABAR-mIPSCs obtained from control (blue, shown in A) and *Sst-Cre x Tsc1*^*fl/fl*^ (red, shown in B) mice, bottom, normalization and superposition of the above averaged GABAR-mIPSCs. Scale bars: 4 pA, 20 ms. **k**-**l** Pooled data showing no significant difference in averaged GABAR-mIPSC rise time (K, Control, 1.83 ± 0.2 ms vs. *Sst-Cre x Tsc1*^*fl/fl*^, 1.75 ± 0.21 ms, *P* = 0.79) and decay time (L, Control, 14.45 ± 0.66 ms vs. *Sst-Cre x Tsc1*^*fl/fl*^, 14.16 ± 1.45 ms, *P* = 0.85) between the mutant and control animals. For pooled data, unpaired two-tailed *t-*test was used, the open diamonds represent individual cells and the filled diamonds are means of the experiments in groups, *n* = 7/1 for control and *n* = 9/2 for *Sst-Cre x Tsc1*^*fl/fl*^ groups

While PV (+) cells terminate their axons to the soma of pyramidal neurons, SST (+) interneurons connect with distal segments of apical dendrites [23, 33]. We generated SST (+) cell-specific *Tsc1* knockout mice by crossing *TSC1*^*fl/fl*^ and *Sst*-IRES-*Cre* strains. The knockout mice (*N* = 5) did not exhibit fatal seizures that are reported in *Syn1* x *Cre* x *Tsc1*^*fl/fl*^ mouse strain [42, 46]. Similarly to *Pv-Cre* x *Tsc1*^*fl/fl*^ mice, *Sst-Cre* x *Tsc1*^*fl/fl*^ animals did not exhibit dorsiflexion of the tail (Straub tail), humped posture, and hindlimb clasping. However, weight growth of *Sst-Cre* x *Tsc1*^*fl/fl*^ mice was significantly lower throughout development (Fig. 4b). Next, we recorded AMPAR-mEPSCs and GABAR-mIPSCs from nine neurons in two *Sst-Cre* x *Tsc1*^*fl/fl*^ mice and seven neurons in one *Tsc1*-floxed without *Cre* mouse (Fig. 6a and b) and observed no significant differences in the amplitude or frequency of the mEPSCs (Fig. 6c and d; *p* = 0.28 and 0.66, respectively). We also analyzed kinetics of AMPAR-mEPSCs (Fig. 6e-g), and both the rise time (Fig. 6f: *p* = 0.82) and decay time (Fig. 6g: *p* = 0.74) were comparable between the two strains. Both amplitde and frequency of mIPSCs were similar between *Sst-Cre* x *Tsc1*^*fl/fl*^ and *Tsc1*-floxed without *Cre* neurons (Fig. 6 h and i; p = 0.74 and 0.97, respectively). Analyses of GABAR-mIPSC kinetics (Fig. 6j-l) showed no significant differences in rise time (Fig. 6k: *p* = 0.79) and decay time (Fig. 6l: *p* = 0.85).

These results suggest that connection between SST (+) interneuron and layer II/III pyramidal neurons does not account for E/I imbalance by itself in the cortical circuit of TSC.

## Discussion

The present study has examined a synaptic substrate of hyperexcitability in TSC using biochemistry and ex vivo electrophysiology. In *Syn1-Cre* x *Tsc1*^*fl/fl*^ mice, we have found a reduction in GABAergic synapse formation. Based on the decrease in GABAR-mIPSC frequency but not in amplitude, we anticipated the vulnerability of synaptic connections between pyramidal neurons and a specific interneuron subtype. However, neither *Pv-Cre x Tsc1*^*fl/fl*^ nor *Sst-Cre x Tsc1*^*fl/fl*^ strains show an alteration in GABAR-mIPSCs. These results suggest two possibilities: (1) an inhibitory cell type other than these two major types are involved in hyperexcitability of a local cortical circuit in TSC, or (2) the lack of the *Tsc1* gene in inhibitory neuronal progenitor cells during neurogenesis affects multiple interneuron subtypes and is accountable for hyperexcitability.

### E/I balance in TSC and other models of neurodevelopmental disorders

The E/I imbalance has been implicated in both epilepsy and autism [7–12]. Although seizures can arise from cortical tubers in TSC, recent studies indicate that grossly normal cortical circuit outside of the tuber can be a source of epilepsy [47, 48]. Aberrant synapse formation and function are also well-documented in animal models of TSC. For example, previous studies documented morphological changes of neurons including axonal growth [49, 50], and dendritic spine pruning [51], which account for abnormal connectivity. *Tsc2* heterozygous mutant mice have impaired L-LTP and long-term memory [17]. *Tsc1*-suppressed neurons with RNA interference have an increase in AMPAR currents [18], and removal of the floxed *Tsc1* gene using an AAV that contains Cre recombinase cDNA diminished metabotropic glutamate receptor-dependent LTD in the hippocampus [20].

We find decreased GABAR-mIPSC frequency in the *Syn1-Cre* x *Tsc1*^*fl/fl*^ mice which are deleted with the gene in all neurons [42, 46]. This pan-neuronal mouse strain also exhibits reductions in both total and surface expressions of GABA_A_R-α1 subunit. Our results are in line with previous reports in mouse and human brains. Specifically, hippocampal neurons in *CamkIIα-Cre* x *Tsc1*^*fl/fl*^ mice also show a reduction in mIPSCs but normal properties of mEPSCs [21]. Further, a reduction in GABAA receptor subunit and benzodiazepine binding has been shown in tissues from TSC patients [52, 53]. Together, these findings suggest that hyperexcitability in TSC results from a reduction in GABAergic inputs on the pyramidal neuron.

### Preserved E/I balance of pyramidal cell in both PV(+) and SST(+) interneuron-specific *Tsc1* knockout mice

In the cortical circuit, interneurons are remarkably heterogeneous and form proper connections during development [22–27, 32, 54]. Different inhibitory neuron subtypes connect with specific subcellular compartments of pyramidal cells, resulting in highly specialized computations of cortical activity. Recent advances in interneuron-specific-Cre mouse strains enable dissecting synaptic functions of each cell-type [28, 29]. For example, PV (+) and SST (+) cells play complementary roles in computing sensory inputs [34–37]. Accordingly, one can assume that malfunctioning of a specific GABAergic input corresponds to particular neurological and psychiatric symptoms [25, 55, 56].

Circuit analyses using cell-type specific mutant mice have been adopted to study the specific synaptic connection in a local brain circuit that underlies hyperexcitability [43, 44]. For example, a mouse model of Rett syndrome showed seizures when *Mecp2* was removed from all interneurons [57]. Furthermore, analyses of cell-type specific knockout mice indicate that the two interneuron subtypes play distinct roles; *Mecp2* deletion in PV(+) neurons causes motor, sensory, memory, and social deficits while the gene deletion in SST (+) neurons causes seizures and stereotypies [38]. Interestingly, removal of MeCP2 from forebrain excitatory neurons also disrupts GABAergic inhibition of the excitatory cells and results in spontaneous seizures [58]. These studies indicate inhibitory synapses onto the pyramidal neuron as a synaptic substrate of hyperexcitability.

Another example is the autosomal-dominant SCN1A sodium channel mutation that underlies Dravet syndrome, a severe epileptic encephalopathy that begins in childhood [59]. In constitutive *Scn1a*^−/+^ mice, the excitability of both PV- and SST-positive cells is reduced, suggesting that an E/I imbalance occurs in both interneuron subtypes [60]. Moreover, selective disruption of the *Scn1a* gene in PV-positive [41, 61] or SST-positive [62] cells increased susceptibility to seizures.

We asked whether a specific interneuron-type is critical for hyperexcitability in the cortical circuit of TSC and initially focused on PV (+) cells. However, we find both AMPAR-mEPSCs and GABAR-mIPSCs are normal in layer 2/3 pyramidal cells of the *Pv-Cre* x *Tsc1*^*fl/fl*^ mouse visual cortex. One caution is that the Cre expression becomes complete around P60 in *Pv-Cre* mice [40, 63] although younger animals express adequate amounts of Cre-recombinase in the cortex around P 30 [64–66]. While *Pv-Cre* x *Tsc1*^*fl/fl*^ mice lived beyond P 60 without seizures, *Syn1-Cre* x *Tsc1*^*fl/fl*^ mice typically start dying by P 20 and do not survive till P60. Therefore, we have analyzed age-matched animals around P30 to minimize age-related biological variables. Interestingly, we found a small but significant change in the mIPSC decay time of *Pv-Cre* x *Tsc1*^*fl/fl*^ mouse pyramidal cells (Fig. 5l). GABA_A_R-α1 subunit is expressed in both soma and dendrites and is accountable for a developmental decrease in the GABAR-mIPSC decay time [67]. One possibility is *Pv-Cre* x *Tsc1*^*fl/fl*^ mouse have pyramidal cells have an alteration in GABAR subunit composition that leads to a delayed developmental subunit switch or pathological receptor regulation. However, further study is needed.

*Sst-Cre* x *Tsc1*^*fl/fl*^ mice also exhibit intact properties of both AMPAR-mEPSCs and GABAR-mIPSCs. These results rule out two major groups of interneurons as the origin of hyperexcitability in the local circuit of *Syn1-Cre* x *Tsc1*^*fl/fl*^ mouse visual cortex. On the other hand, *Syn1-Cre* x *Tsc1*^*fl/fl*^ mice also have reductions in both total and surface expressions of the GABA_A_R-α1 subunit, which is accountable for electrophysiological hyperexcitability of pyramidal neurons. Our results are in line with a study of pyramidal-neuron-specific *CamkIIα-Cre* x *Tsc1*^*fl/fl*^ mouse, which also shows a reduction in GABAR-mIPSCs but not in AMPAR-mEPSCs [21]. Thus, one possibility is that altered GABAR expression in pyramidal neurons is the primary source of hyperexcitability in TSC.

However, other studies suggest that hyperexcitability in TSC arises from a functional defect in interneurons themselves. For example, *Dlx5/6-Cre* x *Tsc1*^*fl/fl*^ mice targeting ventral neural progenitor cells that give rise interneurons in the neocortex and hippocampus exhibited impaired postnatal growth with approximately 50% dying prematurely [68]. While these mice did not have spontaneous seizures, they had a lower threshold to flurothyl-induced myoclonic jerks. Intriguingly, this conditional knockout strain exhibit a 40% reduction of NPY (+) interneurons and 33% reduction of CR (+) interneurons in the neocortex as compared with littermate controls while no alterations were seen in subtypes positive for PV, SST, or vasoactive intestinal peptide (VIP). There is also a study focusing on a different subset of cortical GABAergic interneuron progenitors. This work characterized the *Nkx2.1-Cre* x *Pten* ^*fl/fl*^ mouse, which also exhibits mTOR hyperactivity similarly to *Tsc1* knockout mice, have a preferential reduction in SST (+) neurons relative to PV (+) cells and show deficits in social behavior [69]. Interestingly, PV (+) interneurons have ectopic projections in layer 1, and the target pyramidal neurons have increased IPSC frequency. Despite the difference in their observations, one implication for interpreting our results is that hyperexcitability in TSC may be derived from interneuron progenitor cells that give rise to multiple inhibitory neuron subtypes. Another possibility is that an inhibitory neuron subtype other than PV- and SST-positive cells may be responsible for E/I imbalance in TSC. Another aspect is that mouse models of various neurogenetic diseases exhibit dissimilar involvement of excitatory and inhibitory neurons, reflecting idiosyncratic presentations of symptoms and their onsets.

We have found a reduction in inhibitory inputs to pyramidal neurons in pan-neuron-specific *Tsc1* knockout mice. Our findings corroborate previous studies and support the rationale for the use of GABA uptake inhibitor vigabatrin to treat epilepsy associated with TSC, such as infantile spasm. While our analyses of *Pv-Cre* x *Tsc1*^*fl/fl*^ and *Sst-Cre* x *Tsc1*^*fl/fl*^ mice did not reveal evidence for E/I imbalance in pyramidal cells of the layer 2/3 visual cortex, further studies are needed to disentangle pathological connectivity underlying hyperexcitability in TSC. Identification of the specific cell type(s) that is responsible for epilepsy and cognitive deficits associated with TSC will facilitate developing a novel therapeutics.

## Abbreviations

AMPAR: α-amino-3-hydroxyl-S-methylisoazole-4-propionate receptor
AMPAR-mEPSCs: AMPAR-mediated miniature excitatory postsynaptic currents
E/I balance: Excitation/inhibition balance
GABA_A_R-α1: Gamma-aminobutyric acid type A receptor subunit alpha-1 subunit
GABAR: Gamma-aminobutyric acid receptor
GABAR-mIPSCs: GABAR-mediated miniature inhibitory postsynaptic currents
L-LTP: Late long-term potentiation
mTOR: Mammalian target of rapamycin
PV: Parvalbumin
SST: Somatostatin
TSC: Tuberous sclerosis complex
